# A first case report of clinical response to targeted therapy in a patient with primary myoepithelial carcinoma of the lung harboring EGFR exon 19 deletion

**DOI:** 10.1186/s13000-020-00986-0

**Published:** 2020-06-06

**Authors:** Xiaohong Xie, Xinqing Lin, Ming Liu, Yinyin Qin, Ming Ouyang, Shiyue Li, Yingying Gu, Shuyin Chen, Jianxing Xiang, Chengzhi Zhou

**Affiliations:** 1 Department of Pulmonary and Critical Care Medicine, The First Affiliated Hospital of Guangzhou Medical University, Guangzhou Institute of Respiratory Health, State Key Laboratory of Respiratory Disease, National Clinical Research Center for Respiratory Disease, 151 Yanjiang Road, Guangzhou, 510120 China; 2 Department of Respiratory Pathology, The First Affiliated Hospital of Guangzhou Medical University, Guangzhou Institute of Respiratory Health, State Key Laboratory of Respiratory Disease, National Clinical Research Center for Respiratory Disease, Guangzhou, 510120 China; 3 Burning Rock Biotech, Guangzhou, 510300 China

**Keywords:** Lung, Myoepithelial carcinoma, Salivary gland type tumor, EGFR 19del, Icotinib

## Abstract

**Background:**

Primary myoepithelial carcinoma of the lung is a rare subtype in lung cancer. Comprehensive molecular profiling of myoepithelial carcinoma of the lung is absent, neither was clinical evidence of targeted therapy available for this disease. Therefore, the optimal treatment regimen of this tumor needs to be established.

**Case presentation:**

Here we present a case of a 68-year-old patient with stage IVB primary myoepithelial carcinoma of the lung who harbored EGFR exon 19 deletion and KRAS mutation and underwent icotinib targeted therapy, achieving partial response (PR) with progression free survival (PFS) of 3 months.

**Conclusion:**

To our knowledge, this study describes the first documented case of primary myoepithelial carcinoma lung cancer patient harboring EGFR exon 19 deletion and KRAS mutation, and showed clinical efficacy of epidermal growth factor receptor tyrosine kinase inhibitors (EGFR-TKI) treatment in this patient.

## Introduction

Myoepithelial Carcinoma of the lung is a rare subset of primary salivary-type tumors, accounting for less than 1% in all lung tumors [1]. It was first described in 1975 by Stromeyer et al., and modified by Dardick et al., with the histopathologic guidelines in 1995 [2]. It seems that due to the lack of sufficient knowledge and diagnostic criteria for this tumor in the past, the incident rate of this tumor may be underestimated [3].

Myoepithelial carcinoma usually occurs in middle-aged adults, the average age at diagnosis is around 55 years old. Although it mainly occurs in the parotid gland, it can also be found in primary sites other than the salivary glands, such as lung, skin and soft tissue [4–6]. The molecular profiling of these tumors has not been systematically studied. EGFR mutation is very common in lung adenocarcinoma, but it has not been reported in this tumor owing to the rarity of the disease and lack of attempts to characterize it molecularly. Therefore, it is unknown whether targeted therapies such as EGFR-TKI could achieve similar response in primary myoepithelial carcinoma as in lung adenocarcinoma.

## Case report

**Fig. 1:**
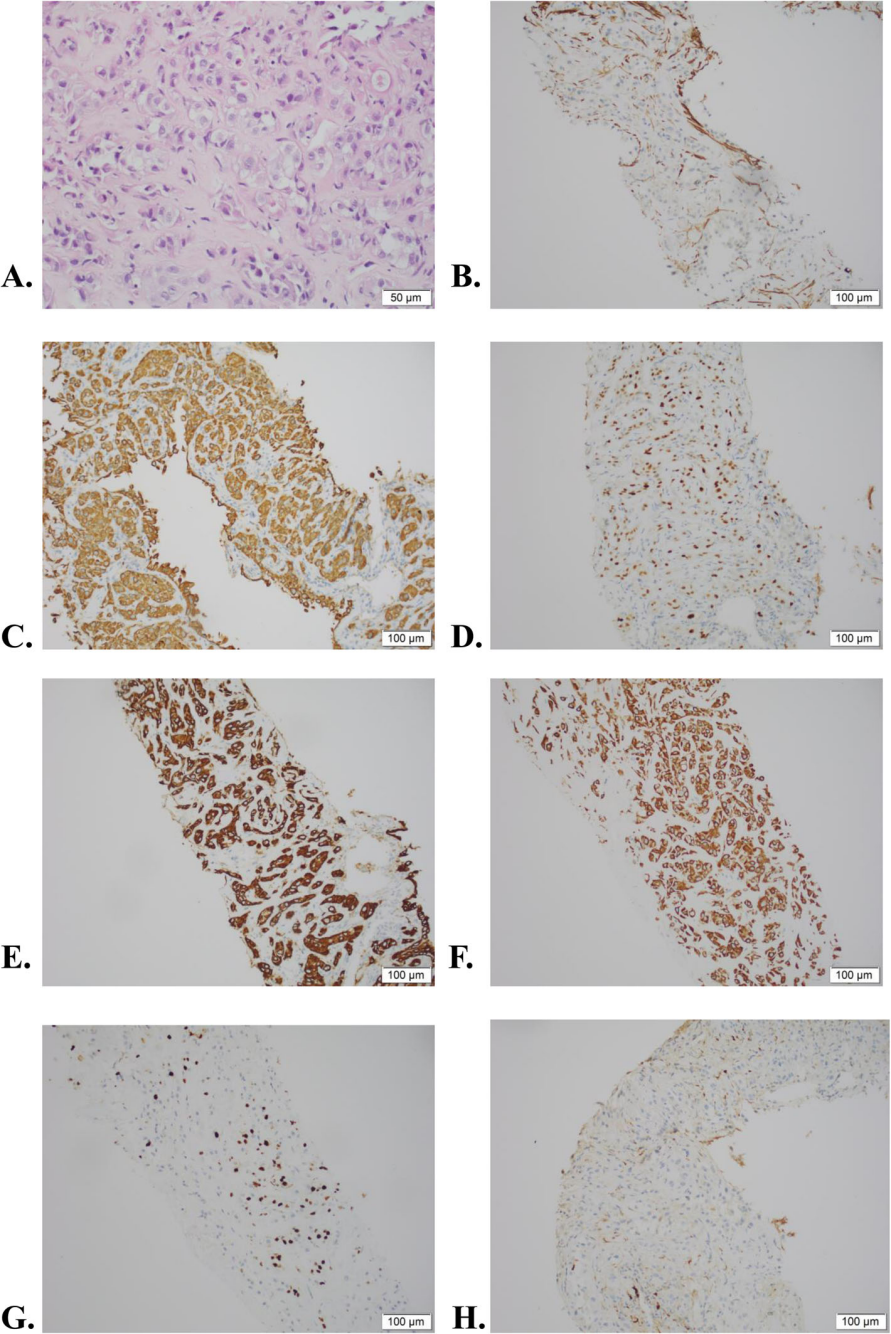
Histologic features of primay myoepithelial carcinoma of the lung. **a** Hematoxylin-eosin (HE) stain (magnification X400): The cancer cells are arranged like small nests with many hyaline substances in the stroma; **b**-**h**. Immunohistochemical staining of Calponin (**b**), CK (**c**), P63 (**d**), CK7 (**e**), CK5/6 (**f**), Ki67 (**g**), S-100 (**h**) (magnification X200)

A 68-year-old man presented to our institution for the treatment with cough and chest pain which already last for 4 months. He had a medical history of chronic gastritis for 3 years, and he was a smoker (30 cigarettes/day) for 40 years. He denied other symptoms like nausea, fever, vomiting or shortness of breath. Electrocardiography shows T wave changes and echocardiography showed moderate tricuspid regurgitation. The left ventricular diastolic pressure decreased. Routine laboratory examinations found carcinoembryonic antigen (CEA) at 4.7 ng/mL (normal range, < 5 ng/mL) within normal limits, but increased level of Prostate-specific antigen (PSA) (5.1 ng/mL; normal range, < 4.4 ng/mL), cancer antigen 125 (CA-125) (41.7 U/mL; normal range, < 35 U/mL), and carbohydrate antigen 19–9 (CA19–9) (53.4.1 U/mL; normal range, < 27 U/mL) in the serum. Furthermore, the fiberoptic bronchoscopic biopsy sample was fixed with 10% neutral formalin, embedded in paraffin, routinely prepared, stained with hematoxylin-eosin, and observed under light microscope. Streptavidin-perosidase method was used for immunohistochemistry. The Immunohistochemical staining results showed that the tumor cells were positive for CK(+), P63(+), CK7(+), CK5/6(+), Ki67(+), S-100(+), Calponin(+), while negative for P40, NapsinA, TTF1, WT-1 and CR (Fig. 1).

Based on the radiopathological result, he was diagnosed with stage IVB myoepithelial carcinoma accompanied by metastases in the left pulmonary hilum, mediastinal lymph nodes, and cervical vertebra. Besides, there was no measurable tumor observed from PET-CT along his salivary gland. His Eastern Cooperative Oncology Group (ECOG) performance status was 1, and his vital signs were normal.

**Fig. 2:**
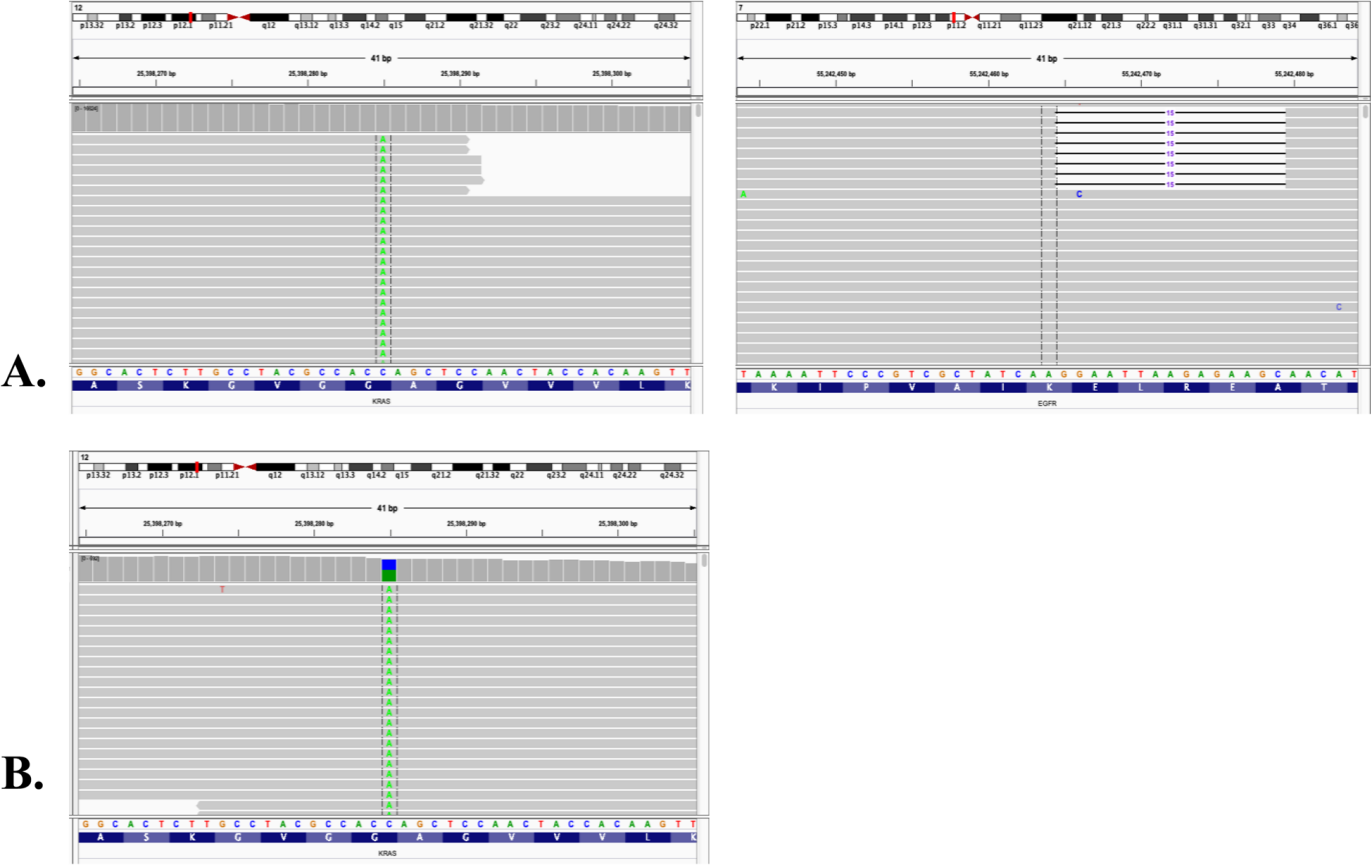
The Intergrative Genomics Viewer (IGV) screenshots displayed the reads from ctDNA sequencing and revealed the harboring of EGFR exon 19 deletion [NM_005228.3(EGFR):c.2235_2249del(p.Glu746_Ala750del)] and KRAS mutation [NM_033360.3(KRAS):c.34G > T(p.Gly12Cys)] from plasma (**a**) and tissue (**b**) samples

**Fig. 3:**
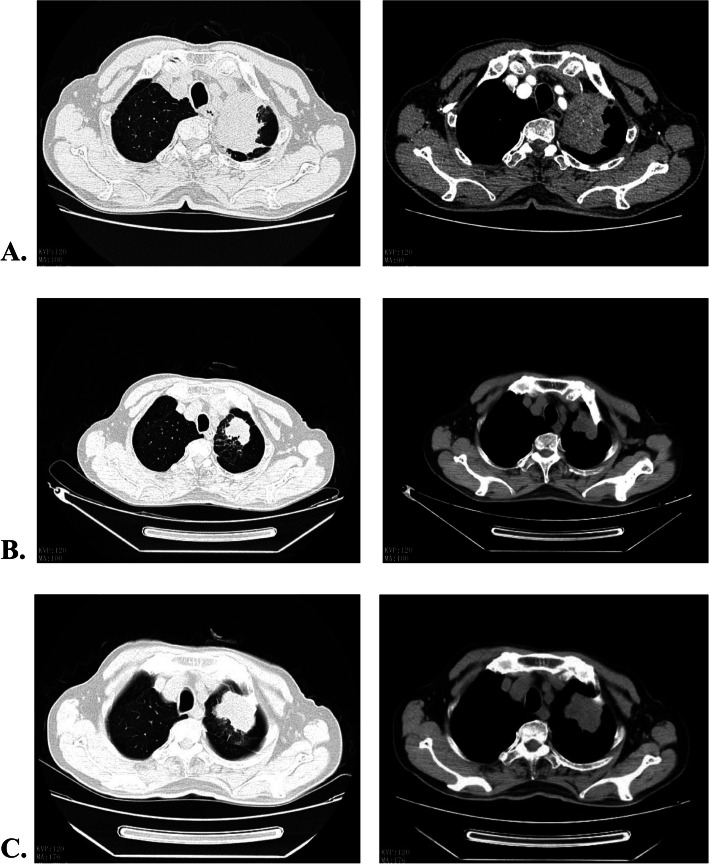
Computed tomography scans of the patient showing the tumor responses to icotinib target therapy. **a** Scans at the baseline when diagnosed with primay myoepithelial carcinoma of the lung on Jul. 17, 2019 (tumor diameter of 6.2 cm × 4.7 cm). **b** Scans after icotinib regimen, with the tumor diameter of 4.2 cm × 3.5 cm and disease evaluated as PR on Sep. 07, 2019. **c** Scans during disease progression with tumor diameter of 4.8 cm × 4.7 cm on Oct. 07, 2019

Furthermore, the biopsy samples were tested by targeted next generation sequencing (NGS) with a 168-gene cancer panel (Burning Rock Biotech, Guangzhou, Guangdong, China). The NGS sequencing results revealed EGFR exon 19 deletion (p.E746_A750del; 0.04%) and KRAS mutation (p.G12C; 0.15%) from plasma sample, and only the KRAS mutation (p.G12C; 53.18%) from needle biopsy tissue sample of the lung (Fig. 2). Subsequently, he was treated with EGFR-TKI icotinib at 125 mg orally thrice daily from July 25th 2019. After 40 days, the patient came back for reexamination of chest CT. The radiography showed a significant reduction in his primary lung lesion (6.2 × 4.7 cm vs 4.2 × 3.5 cm). There was significant improvement in clinical symptoms including chest pain. He achieved partial response (PR) under the criteria of Response Evaluation Criteria In Solid Tumors (RECIST) 1.1 (Fig. 3). Unfortunately, the disease progressed after 2.5 months of EGFR-TKI treatment. The magnetic resonance imaging (MRI) revealed disseminated bone metastases and brain atrophy. On Oct 31st 2019, the patient passed away due to multiple organ failures with an overall survival (OS) of 3 months.

## Discussion

The most common practice to manage localized myoepithelial carcinoma is by performing surgical excision with or without adjuvant radiation therapy [7]. However, myoepithelial carcinoma has a high tendency of local recurrences and metastases that warrants close clinical follow-up. After developing into advanced disease, systemic therapy plays an essential role in prolonging survival. Although chemotherapy was the most frequently used systemic therapy, some studies showed that this tumor is not sensitive to chemotherapy [8]. And there is limited evidence of targeted therapy and immunotherapy. Our case is unique because we presented the first clinical evidence of EGFR-TKI targeted therapy for myoepithelial carcinoma.

EGFR mutation is very common in lung cancer patients. But pulmonary myoepithelial carcinoma is a rare subtype of lung carcinoma; there is no previous report of myoepithelial carcinoma patient harboring EGFR mutation. To the best of our knowledge, this is the first time that a myoepithelial carcinoma patient was reported to carry EGFR mutation (exon 19 deletion) and achieve PR after EGFR-TKI therapy.

Previous studies in lung cancer suggested that KRAS mutation is a poor prognostic marker for response to EGFR TKI treatment [9–11]. Especially the KRAS subtype of G12C mutation showed significant shorter PFS and OS than other KRAS mutation types [12]. Taken a glance in the BENEFIT study, the lung adenocarcinoma patients with concurrent mutations in EGFR and other oncogenes, including KRAS, had significantly shorter PFS than those with EGFR mutations only (13.2 months VS 4·7 months, HR 2.66, 95% CI 1.58–4.49; *p* = 0.0003) [11]. Therefore, the relatively short PFS time in our myoepithelial carcinoma patient may be due to the accompanying gene aberrances. Also, clonal heterogeneity might explain this relatively short PFS outcome. From the molecular tests, EGFR mutation was only found in liquid biopsy with very low concentration. It indicated that EGFR might not constitute the major clone of the tumor, and rather the subclone which was missed out by needle biopsy. Instead, KRAS was considered as the dominant driver mutation in this case, which might explain the reduced efficacy achieved by EGFR targeted therapy.

In brief, this case provides evidence of the potential use of targeted therapy in primary myoepithelial carcinoma of the lung. In addition, it highlights the importance of NGS analysis with the baseline biopsy sample, which may aid in the determination and prognostic prediction of therapeutic choices. We believe that the molecular profiling of myoepithelial carcinoma warrants further investigations, which could help determine whether targeted therapy should be included as a routine therapeutic option in myoepithelial carcinoma.

## Conclusions

In conclusion, we presented a case report demonstrating clinical evidence of the efficacy of icotinib in a patient with advanced myoepithelial carcinoma of the lung who harbored EGFR exon 19 deletion. We also provided evidence that molecular profiling and targeted therapy might be potential critical implications in this rare type of disease.

## Data Availability

Is available upon request from the corresponding author.

## Abbreviations

CEA: Carcinoembryonic antigen
PFS: Progression-free survival
TKI: Tyrosine kinase inhibitor
PR: Partial response
ECOG: Eastern Cooperative Oncology Group
RECIST: Response evaluation criteria in solid tumors
NGS: Next-generation sequencing
OS: Overall survival
